# Altered miRNA expression in canine retinas during normal development and in models of retinal degeneration

**DOI:** 10.1186/1471-2164-15-172

**Published:** 2014-03-01

**Authors:** Sem Genini, Karina E Guziewicz, William A Beltran, Gustavo D Aguirre

**Affiliations:** Department of Clinical Studies-Philadelphia, Section of Ophthalmology, School of Veterinary Medicine, University of Pennsylvania, 3900 Delancey Street, 19104 Philadelphia, PA USA

**Keywords:** Apoptomirs, Canine models, erd, Inherited retinal disorders, Microarray, miRNA expression profiles, prcd, qRT-PCR, rcd1, xlpra2

## Abstract

**Background:**

Although more than 246 loci/genes are associated with inherited retinal diseases, the mechanistic events that link genetic mutations to photoreceptor cell death are poorly understood. miRNAs play a relevant role during retinal development and disease. Thus, as a first step in characterizing miRNA involvement during disease expression and progression, we examined miRNAs expression changes in normal retinal development and in four canine models of retinal degenerative disease.

**Results:**

The initial microarray analysis showed that 50 miRNAs were differentially expressed (DE) early (3 vs. 7 wks) in normal retina development, while only 2 were DE between 7 and 16 wks, when the dog retina is fully mature. miRNA expression profiles were similar between dogs affected with xlpra2, an early-onset retinal disease caused by a microdeletion in *RPGR*ORF15, and normal dogs early in development (3 wks) and at the peak of photoreceptor death (7 wks), when only 2 miRNAs were DE. However, the expression varied much more markedly during the chronic cell death stage at 16 wks (118 up-/55 down-regulated miRNAs). Functional analyses indicated that these DE miRNAs are associated with an increased inflammatory response, as well as cell death/survival. qRT-PCR of selected apoptosis-related miRNAs (“apoptomirs”) confirmed the microarray results in xlpra2, and extended the analysis to the early-onset retinal diseases rcd1 (*PDE6B*-mutation) and erd (*STK38L*-mutation), as well as the slowly progressing prcd (*PRCD*-mutation). The results showed up-regulation of anti-apoptotic (miR-9, -19a, -20, -21, -29b, -146a, -155, -221) and down-regulation of pro-apoptotic (miR-122, -129) apoptomirs in the early-onset diseases and, with few exceptions, also in the prcd-mutants.

**Conclusions:**

Our results suggest that apoptomirs might be expressed by diseased retinas in an attempt to counteract the degenerative process. The pattern of expression in diseased retinas mirrored the morphology and cell death kinetics previously described for these diseases. This study suggests that common miRNA regulatory mechanisms may be involved in retinal degeneration processes and provides attractive opportunities for the development of novel miRNA-based therapies to delay the progression of the degenerative process.

**Electronic supplementary material:**

The online version of this article (doi:10.1186/1471-2164-15-172) contains supplementary material, which is available to authorized users.

## Background

The visual process begins in highly specialized photoreceptors (PRs), which are neurons with a complex structure and a unique ability to convert light photons into electrochemical messages. After the initial quantal light absorption by the rhodopsin visual pigments, a signal is generated in the PR and subsequently transmitted through two different synaptic pathways in the outer and inner plexiform layers. The information is then conveyed to higher visual centers by the ganglion cell axons. To subserve this role, a large number of genes are involved in PR specification, differentiation, and maintenance [1]; and mutations in many of these genes impair PR function and viability. Indeed, of the 246 loci that are associated with retinal degeneration in humans, in 206 the disease-causative genes have been identified [2]. Several genes have also been associated with retinal degeneration in animals [3], and at least 24 mutations in 18 genes related to canine retinal degenerations have been identified [4].

Although the number of identified genetic mutations underlying different forms of retinal degeneration is systematically growing, the molecular events and key components that link specific mutations to PR degeneration remain poorly characterized. Multiple pathways, both pro-apoptotic and pro-survival, are associated with PR degeneration [5–9]. Furthermore, epigenetic mechanisms, including miRNA regulation, also play an essential role in the control of the complex visual processes during eye development and disease [10]. miRNAs are small (~20-25 bp), endogenous, non-coding single-stranded regulatory RNA molecules that regulate various cellular functions, including differentiation, proliferation, and cell death/survival (reviewed by [11]). They are expressed in all living organisms in tissue- and developmental stage-specific manner, and are responsible for individual phenotypical variations. miRNAs silence gene expression via cleavage, degradation, and/or translational inhibition of their downstream target mRNAs (reviewed for mammalian miRNAs by [12]). Each miRNA has the potential to regulate multiple different mRNA targets simultaneously, while a given mRNA target might similarly be targeted by multiple miRNAs (reviewed by [13]). The number of known mature miRNAs is currently 30,424 in 193 species and approximately 2,580 have been discovered in humans [14]. Of these, 349 have been linked to 163 different diseases [15].

Recently, the use of miRNA-microarray analysis in several tissues has enabled the identification of altered miRNA transcriptomes during development/aging and disease, including profiles of pathologically altered miRNAs in the eye and retina (reviewed by [16]). Also, it has been shown that miRNA pathways control important steps during the developmental timing of retinogenesis [17], and appear to regulate neuronal differentiation [18]. The use of other technologies (i.e. deep sequencing) has also provided very comprehensive profiles of miRNAs and revealed a complex expression pattern of small RNA in the mouse retina and RPE/choroid [19]. Of particular interest are “apoptomirs” [20], miRNAs that have been shown in many studies to be relevant mediators of cell death signaling [21–23]. Assessment of disease-related miRNAs in human retinal diseases obviously is limited by the availability of appropriately staged tissues from patients having the same disease and causative gene mutation. Notably however, the dog has been widely recognized as an ideal model for a variety of human retinal disease studies, as canine inherited retinopathies result from mutations in disease gene homologues and exhibit comparable phenotypic features, including age of onset and progression [4]. Some models have the advantage of an early and predictable disease course, making the time window for experimentation very short and easily comparable. As such, they are an ideal system in which to determine if miRNAs are associated with PR death and if their involvement is dependent on the specific mutation driving disease.

To identify potential miRNAs associated with PR degeneration, we used the four following canine models: X-linked progressive retinal atrophy 2 (xlpra2), rod cone dysplasia 1 (rcd1), early retina degeneration (erd), and progressive rod-cone degeneration (prcd) that have mutations in *RPGR*, *PDE6B*, *STK38L*, and *PRCD*, respectively. The progression and histopathology in xlpra2, rcd1, and erd are comparable, and characterized by a fast degeneration of the PR cell layer and decreased number of PRs [24–27]. The first two models have mutations in genes that cause human inherited blindness, while no equivalent human disease for erd has been reported yet. In contrast, prcd is a post-developmental, slowly progressive disease where human patients and animal models show disease variation in the presence of the same mutation [28, 29].

For the initial microarray analysis, retinas of dogs affected with xlpra2 were compared to normal samples. To expand the microarray results, we then undertook a qRT-PCR analysis of the expression of selected apoptomirs in the three additional models, rcd1, erd, and prcd.

Our results show that although different mutations trigger the retinal diseases studied, there are commonalities in the miRNA expression pattern that appear to be associated with the PR cell death kinetics.

## Results

### miRNA expression profiles of normal and xlpra2 retinas

**Figure 1:**
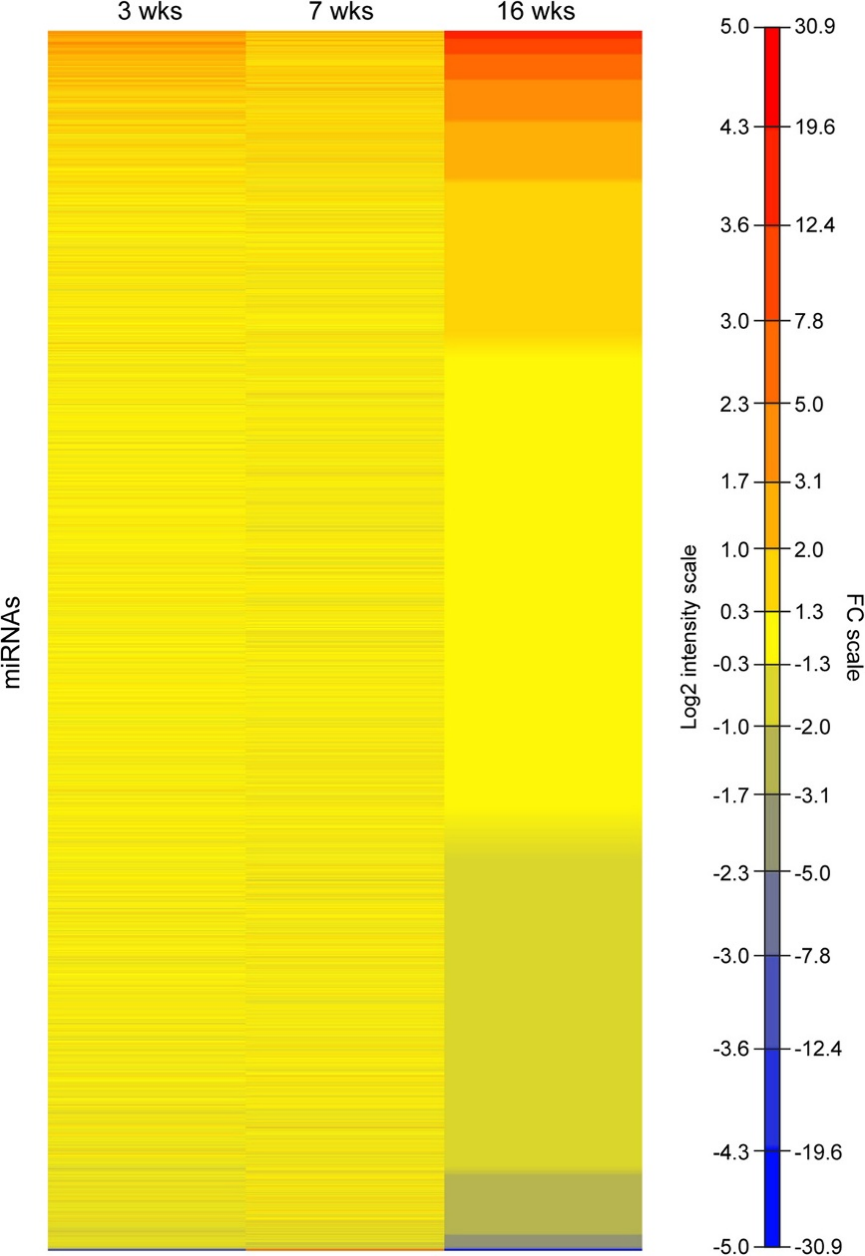
**Heat map representation of all miRNAs present on the microarray.** The heat map illustrates the expression differences of all miRNAs on the microarray between xlpra2-mutants vs. normals at the 3 tested ages (3, 7, and 16 wks). miRNAs are listed from the highest to the lowest fold change difference at 16 wks. The x-axis shows the ages, while the y-axis displays the different miRNAs. The map contains log2 intensity/fold change ratios that are color coded with red corresponding to up-regulation and blue to down-regulation.

We used Affymetrix microarrays to generate comprehensive miRNA expression profiles of retinas from normal and xlpra2-mutant dogs. Retinas were examined at 3, 7, and 16 wks of postnatal age, the time points relevant for detection of developmental and degeneration-related miRNAs [24]. In mutant retinas, the 3 wk time point (*induction* phase) is prior to the beginning of apoptosis and retinal structure is normal. The *execution* phase at 7 wks shows a ~10-15% decrease in PR number and is associated with the peak of cell death. Lastly, at the *chronic cell death* phase (16 wks) the mutant retina shows a sustained but reduced cell death rate and a persistent low-grade degeneration with loss of 40% of the PR layer [24]. A heat map illustrating the expression differences of all miRNAs present on the microarray in xlpra2-mutants compared to normals (log2 and FC ratios) at the 3 ages is shown as Figure 1. Only miRNAs showing FC difference > +/-2 and a Benjamini-Hochberg (BH)-adjusted p < 0.05 were considered significant, and they are detailed in Additional files 1, 2, and 3. The BH procedure was applied to control the false discovery rate, which is the proportion of “discoveries” (significant results) that are actually false positives.

#### Age-dependent miRNA expression changes during development in normal and xlpra2 retinas-within group comparisons

To properly assess the potential variation in miRNA expression within each experimental group, we first characterized the miRNA expression profile during development of normal retinas by comparing the 3, 7, and 16 wks time points. While the retina is still developing at 3 wks, it reaches structural maturation at 7 wks, and at 16 wks it is considered fully developed [26]. Results showed that miRNAs in normal retinas were differentially expressed (DE) at 7 vs. 3 wks (27 up-/23 down-regulated) and 16 vs. 3 wks (42 up-/52 down-regulated) (Additional file 1) thus identifying developmentally regulated differences that occur when the retina is developed (7 and 16 wks) or early in differentiation (3 wks). In contrast, there were limited expression differences between the 16 and 7 wk time points (1 up-/1 down-regulated) (Additional file 1). Thus our results suggest that changes in miRNA expression parallel the structural development of the normal dog retina, with major changes occurring between 3 wks and later time points, and minimal differences occurring between 16 and 7 wks when the retina is fully mature. Although changes in miR-122 expression were not statistically significant due to high variation between biological replicates, it showed the largest FC differences among all miRNAs, being highly down-regulated at 7 compared to 3 wks (FC = -13.2) and 16 wks (FC = -29.6).

In parallel, we examined miRNA expression during development in xlpra2-mutant retinas. A total of 37 (21 up-/16 down-regulated) DE miRNAs were identified at 7 vs. 3 wks, 22 (14 up-/8 down-regulated) at 16 vs. 7 wks, and 56 (38 up-/18 down-regulated) at 16 vs. 3 wks (Additional file 2). Interestingly, while the 2 DE miRNAs (miR-363 and -187) in normal retinas between 16 and 7 wks were not DE in xlpra2 at the same ages, 19 DE miRNAs between 7 and 3 wks (miR-17-5p, -18, -18a, -18b, -29a, -29b-2-star, -29c-star, -34b, -34c, -106a, -106b, -129, -130b, -133a, -133b, -133c, -133d, -211, -363), and 21 between 16 and 3 wks (miR-28, -29a, -29b, -29b-2-star, -29c, -29c-star, -34b, -34c, -34c-3p, -92a-1-star, -130b, -135a-star, -187, -212, -221, -222, -222a, -326, -363, -431, -551a) were DE in both normal and xlpra2 within group comparisons. The identification of these common DE miRNAs indicates that similar mechanisms occur in normal and xlpra2 retinas until structural maturation is completed. At later time points, miRNA profiles in mutant retinas are more variable suggesting that miRNA-related mechanisms that may compromise retinal function are activated between these phases of the disease.

#### miRNA expression changes between normal and xlpra2-mutant retinas

**Figure 2:**
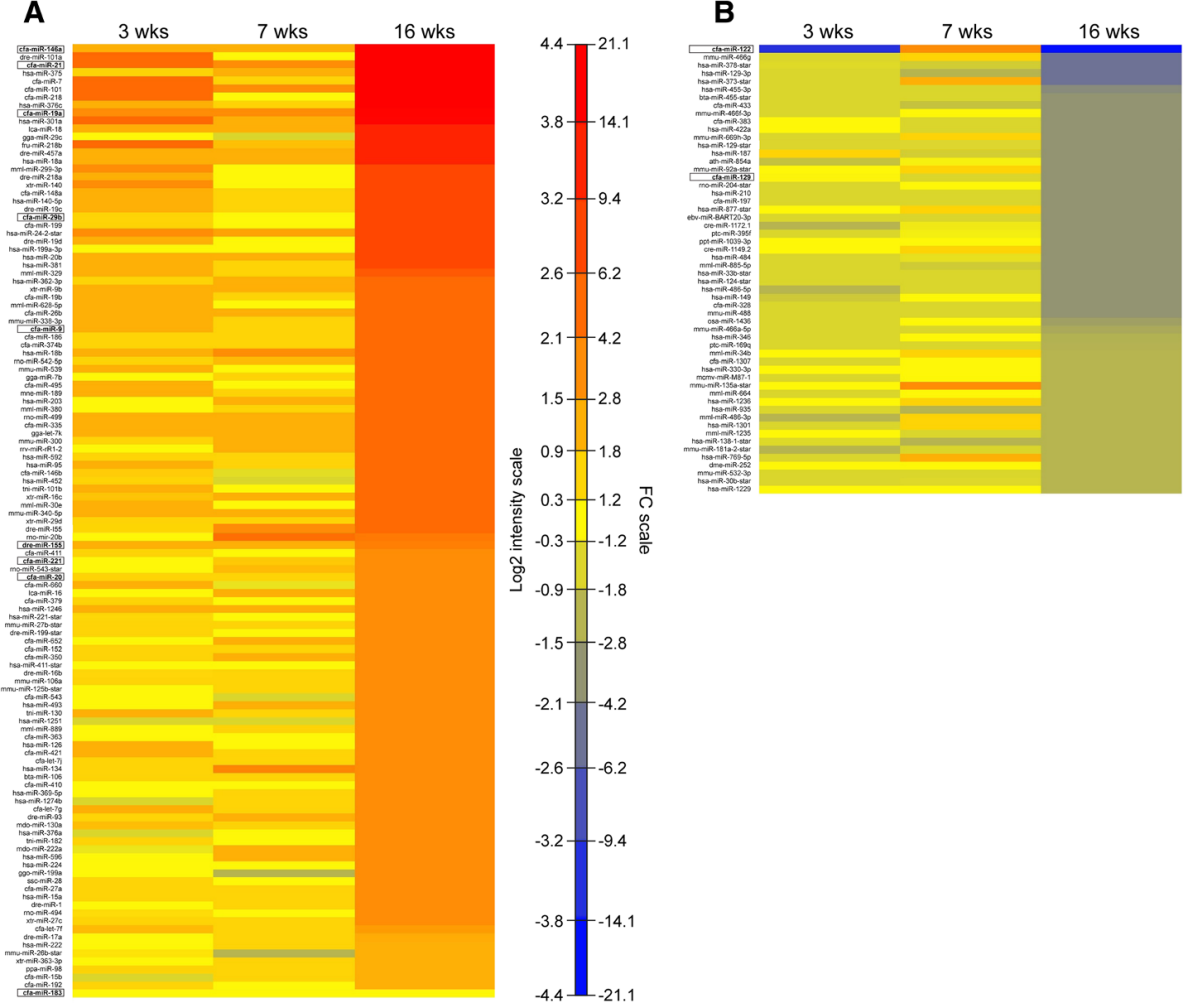
**Heat map representation over time of the DE miRNAs at 16 wks.** The heat map illustrates the fold change differences identified by microarray analysis between xlpra2-mutant and normal retinas at all three ages (3, 7, and 16 wks) for the up-regulated **(A)** and down-regulated **(B)** miRNAs at 16 wks. miR-183 and miR-122 were also tested by qRT-PCR, therefore they were also included in spite of their expression differences being not statistically significant. miRNAs are listed from the highest to the lowest fold change difference at 16 wks. The x-axis shows the time points, while the y-axis displays the DE miRNAs at 16 wks. The map contains log2 intensity/fold change ratios that are color coded with red corresponding to up-regulation and blue to down-regulation. Apoptomirs that were selected for qRT-PCR analysis are boxed and marked in bold.

To identify miRNAs that are associated with the xlpra2-disease process, we directly compared miRNA expression profiles of xlpra2 and normal retinas at 3 disease phases: *induction*-3 wks, *execution*-7 wks, and *chronic cell death*-16 wks. No differences in miRNA expression were found at 3 wks, and only 2 miRNAs were up-regulated in xlpra2-mutants at 7 wks (Additional file 3). Yet at the 16 wk time point, 173 (118 up-/55 down-regulated) miRNAs were DE in xlpra2 compared to normals (Additional file 3). Of the 2 DE miRNAs identified at 7 wks, only miR-155 was also DE at 16 wks. A graphical illustration of all the DE miRNAs at 16 wks is shown in the heat maps, which illustrate the up- (Figure 2A) and down- (Figure 2B) regulated miRNAs at 16 wks, and their expression patterns at the earlier time points in the disease. Some highly up-regulated miRNAs (e.g. miR-146a, -19a, -21, -101) at 16 wks in xlpra2 vs. normals also showed high fold change at early ages, although they were not statistically significant (Figure 2A). Of interest was the irregular expression pattern of miR-122 in xlpra2 compared to normals; although not significant, this miRNA showed the lowest FC differences at 3 and 16 wks, while it was increased at 7 wks (Figure 2B).

These results demonstrate that whereas miRNA expression differences were minimal during either the *induction* or *execution* phases of the disease, i.e. 3 and 7 wks, a substantial number of altered miRNAs were identified during the *chronic cell death* phase. This suggested that miRNAs would not be the initiators of the PR degeneration process but instead may represent co-effectors and/or arise in response to retinal disease progression.

### Functional grouping and target analysis of the DE miRNAs at 16 wks

To further assess the potential functional significance of DE miRNAs during the *chronic cell death* phase of the diseases, we investigated the relationships and common biological functions of the 173 DE miRNAs using the Ingenuity Pathway Analysis (IPA) database. The 7 networks that were significantly associated with the DE miRNAs at 16 wks were: (1) Cancer, Reproductive System Disease, Endocrine System Disorders (23 up- and 3 down-regulated miRNAs, see Additional file 4 for a graphical representation); (2) Connective Tissue Disorders, Inflammatory Disease, Inflammatory Response (11 up- and 10 down-regulated miRNAs); (3) Reproductive System Disease, Connective Tissue Disorders, Inflammatory Disease (13 up- and 5 down-regulated miRNAs); (4) Endocrine System Disorders, Reproductive System Disease, Metabolic Disease (9 up- and 6 down-regulated miRNAs); (5) Reproductive System Disease, Cancer, Respiratory Disease (8 up- and 7 down-regulated miRNAs); (6) Cancer, Gastrointestinal Disease, Hereditary Disorder (14 up-regulated miRNAs); (7) Endocrine System Disorders, Reproductive System Disease, Connective Tissue Disorders (13 up-regulated miRNAs) (Additional file 5). Identified genes and node molecules of particular interest are those with known functional relevance in the retina and/or are related to apoptosis, a hallmark of our disease models. These include *NFkB*, *PARP*, pro-inflammatory cytokines (network 1, Additional file 4), *CREB1* (network 2), *DICER1* (network 3), *PAX6* (network 4), *E2F1*, tretinoin, *VIM* (network 5), *BIRC5*, *SIRT1*, tretinoin (network 6), *IL21*, *Vegf* (network 7).

The 5 IPA biological functions showing the highest association with the misregulated miRNAs were identified and summarized in Additional file 6. Although the DE miRNAs were related to general diseases/disorders including the inflammatory response, of particular interest were DE miRNAs associated with cellular development, cellular growth and proliferation, cell cycle, cell death and survival, and cell-to-cell signaling and interaction. These data suggest involvement of the DE miRNAs in the *chronic cell death* phase and therefore in the progression of the disease. The results also indicate that the DE miRNAs might be related to the inflammatory response, which has been shown to be relevant during retina degeneration in several diseases [30–32].

Finally, to identify potential target molecules that might be directly affected by over-expression of miRNAs, we determined *in silico* common targets of the highly up-regulated miRNAs at 16 wks (FC > 5, Additional file 3). A total of 35 genes were identified (Table 1), including *CREB1*, one of the genes in IPA network 2 that was already associated with the DE miRNAs (Additional file 5).

**Table 1:** **Common gene targets of all the up-regulated (FC > 5) miRNAs identified at 16 wks**

Gene target symbol	Definition	NCBI accession sequences
*FBXO28*	F-box protein 28	NM_015176; AK303381
*BBX*	Bobby sox homolog (Drosophila)	NM_020235; NM_001142568
*SPRED1*	Sprouty-related, EVH1 domain-containing protein 1	NM_152594
*MMAA*	Methylmalonic aciduria (cobalamin deficiency) type A (mitochondrial)	NM_172250
*ST8SIA4*	ST8 alpha-N-acetyl-neuraminide alpha-2,8-sialyltransferase 4	NM_005668
*KCNK10*	potassium channel, subfamily K, member 10	NM_021161; NM_138317
*USP9Y*	Ubiquitin specific peptidase 9, Y-linked	NM_004654
*ALG11*	Asparagine-linked glycosylation 11, alpha-1,2-mannosyltransferase homolog (Yeast)	NM_001004127
*DIEXF*	Digestive organ expansion factor homolog (Zebrafish)	NM_014388; AK314061
*RORA*	RAR-related orphan receptor A	NM_002943; NM_134260
*PHACTR2*	Phosphatase and actin regulator 2	NM_014721; NM_001100164
*SNTB2*	Syntrophin, beta 2 (dystrophin-associated protein A1, 59 kDa, basic component 2)	NM_006750
*BCAT1*	Branched chain amino-acid transaminase 1, cytosolic	NM_005504; AK128527
*PAG1*	Phosphoprotein associated with glycosphingolipid microdomains 1	NM_018440
*CREB1*	cAMP responsive element binding protein 1	NM_004379; NM_134442
*PTAR1*	Protein prenyltransferase alpha subunit repeat containing 1	NM_001099666
*TFCP2L1*	Transcription factor CP2-like 1	NM_014553; AL137740
*SLC1A2*	Solute carrier family 1 (glial high affinity glutamate transporter), member 2	NM_004171; U01824
*ACVR2B*	Activin A receptor, type IIB	NM_001106; BC096245
*ST8SIA3*	ST8 alpha-N-acetyl-neuraminide alpha-2,8-sialyltransferase 3	NM_015879
*UBN2*	Ubinuclein 2	NM_173569
*ADCY1*	Adenylate cyclase 1 (brain)	NM_021116
*DNAH14*	Dynein, axonemal, heavy chain 14	NM_001373
*SSR1*	Signal sequence receptor, alpha	NM_003144; CR599599
*FUT9*	Fucosyltransferase 9 (alpha (1,3) fucosyltransferase)	NM_006581
*SCAI*	Suppressor of cancer cell invasion	NM_173690; NM_001144877
*CDK6*	Cyclin-dependent kinase 6	NM_001259; NM_001145306
*TNRC6B*	Trinucleotide repeat containing 6B	NM_015088; NM_001024843
*LOC728264*	Homo sapiens cDNA FLJ44517 fis, clone UTERU3002667	AK126481
*CBX5*	Chromobox homolog 5	NM_012117; NM_001127321
*NSL1*	Kinetochore-associated protein NSL1 homolog	NM_015471; AK303250
*KSR2*	Kinase suppressor of ras 2	NM_173598
*NEAT1*	Nuclear paraspeckle assembly transcript 1	NR_028272
*SH3TC2*	SH3 domain and tetratricopeptide repeats 2	NM_024577; AB075865
*TSIX*	X (inactive)-specific transcript, antisense	NR_003255

The complete list of DE miRNAs identified at 16 wks between xlpra2 and normal retinas is shown in Additional file 3. The 35 common gene targets are reported with their symbols, definitions, and NCBI accession numbers.

### qRT-PCR analysis of selected apoptomirs to validate the microarray data and expand the results to additional canine models

Based on the functional analyses and phenotypical evidence of PR cell death, we used qRT-PCR of 11 selected DE apoptomirs to validate the microarray results (Figure 2). The main functions of the selected apoptomirs were anti-apoptotic (miR-9, -19a, -20, -21, -155, -183, -221), pro-apoptotic (-122, -129), or miRNAs with dual anti- and pro-apoptotic properties (-29b, -146a) (for references see Additional file 6). In addition, we characterized changes in apoptomir expression in additional canine models: rcd1, erd, and prcd. Although there is no observed peak of cell death in the prcd disease, PRs begin to degenerate first in the inferior and then in the superior region of the retina after 25 wks, at which time the ERG is altered. This disease is of particular interest for epigenetic control because human and animal patients show phenotypical variations in the presence of the same mutation, both in severity and in affected retinal regions (superior/inferior) [33]. Thus, the identification of miRNAs as prcd modulators that influence the disease phenotypes is relevant to provide insights into this specific disease mechanism.

#### Expression changes of apoptomirs during development-within group analysis

**Figure 3:**
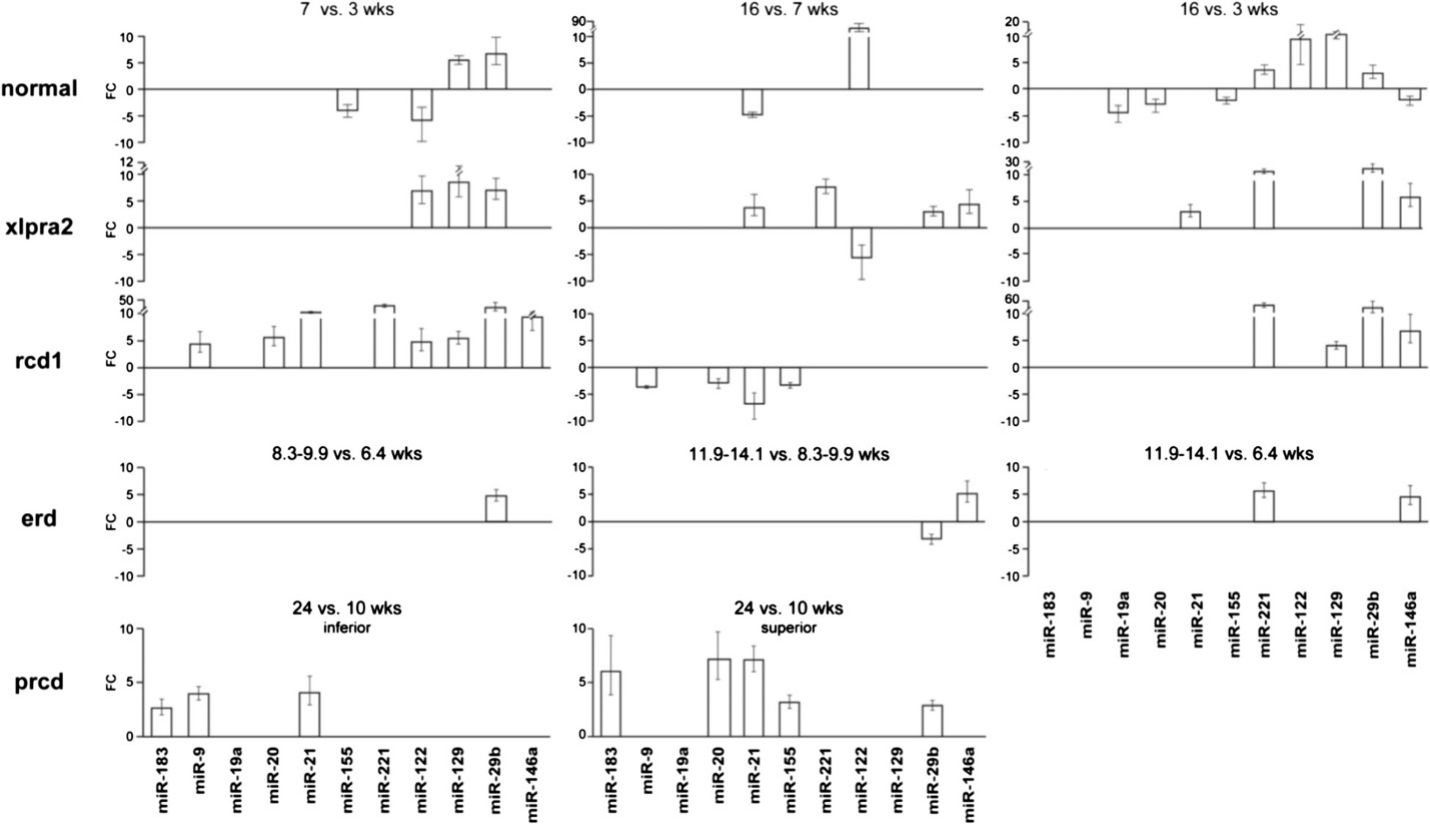
**Expression changes of DE apoptomirs during development.** Significant FC differences as measured by qRT-PCR are reported at 7 vs. 3, 16 vs. 7, and 16 vs. 3 wks in normal, xlpra2, and rcd1; at 8.3/9.9 vs. 6.4, 11.9/14.1 vs. 8.3/9.9, and 11.9/14.1 vs. 6.4 wks in erd; and at 24 vs. 10 wks in inferior and superior retinas of prcd-mutants. Bars show SD of biological triplicates. Only DE apoptomirs were displayed.

We initially analyzed the expression of apoptomirs during development in normal retinas. miR-155 was up-regulated at 3 compared to 7 and 16 wks, while miR-129 and miR-29b showed the opposite trend. miR-122 was highly up-regulated at 16 wks compared to the other two ages, and was down-regulated at 7 vs. 3 wks. Other down-regulated miRNAs included miR-21 at 16 vs. 7, and miR-19a, -20, -221, -146a at 16 vs. 3 wks (Figure 3). In the xlpra2 mutant retinas, miR-122 was up-regulated at 7 compared to 3 or 16 wks. Expression of additional apoptomirs increased at later ages, e.g. miR-129 and -29b at 7 vs. 3 wks, and miR-21, -221, -29b, -146a at 16 wks compared to early time points (Figure 3).

With few exceptions, the qRT-PCR and the microarray data within groups were in agreement. Although FC differences were similar in both analyses, expression of miR-122 only achieved statistical significance in the qRT-PCR analysis. Also miR-21, -29b at young ages, and miR-146a were found to be DE by qRT-PCR but not microarray analysis. The BH-adjustment of the p-value likely was responsible for these differences, as it was applied in the microarray analysis and not in qRT-PCR.

The qRT-PCR results were different during development in rcd1, erd, and prcd retinas. In rcd1, the expression of miR-19a, -155, and -183 did not vary in any of the ages. The remaining miRNAs had a peak of expression at 7 wks; they were all up-regulated vs. 3 wks and miR-9, -20, -21, -155 were also up-regulated vs. 16 wks (Figure 3). Thus, expression changes in rcd1 were similar to those in xlpra2, although in the latter disease they appeared to be slightly delayed (i.e. between 16–7 wks); these differences reflect the more severe and faster disease course of rcd1. In erd retinas, apoptomir expression changes were minimal; miR-221 was up-regulated at 11.9-14.1 vs. 6.4 wks, while miR-29b and -146a showed peaks of expression at 8.3-9.9 and 11.9-14.1 wks, respectively (Figure 3). In prcd inferior and superior retinas, 3 and 5 miRNAs, respectively, were up-regulated at 24 vs. 10 wks. Two of them (miR-183 and -21) were altered regardless of the retinal location (Figure 3).

The miRNA expression profiles reflect the cell death kinetics and the phenotypical changes observed for the 3 early-onset diseases, which show that rcd1 is a very aggressive disease with morphological changes occurring early in life, while xlpra2 and erd are more moderate.

#### Expression changes of apoptomirs between mutant and normal retinas

**Figure 4:**
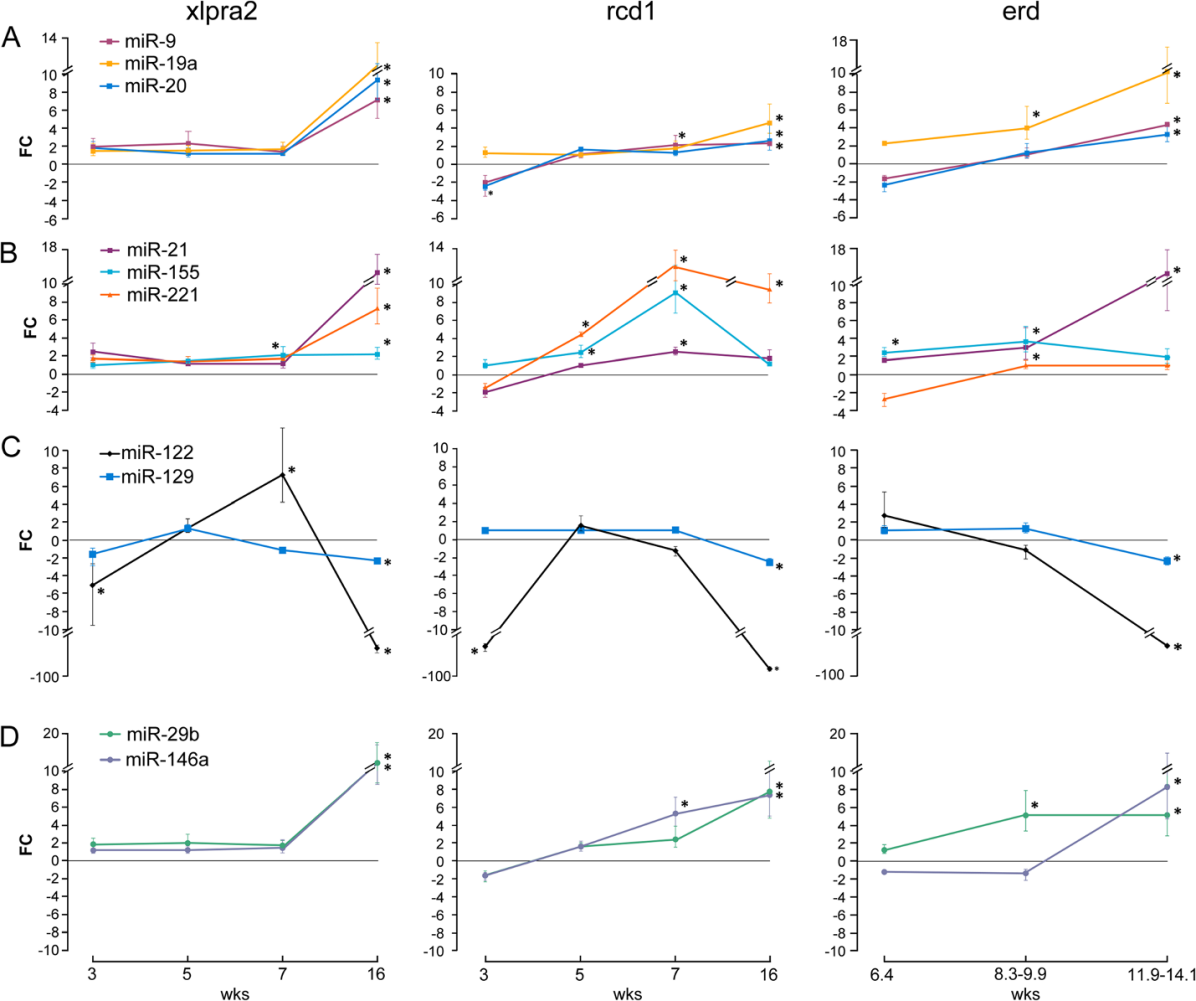
**Expression changes of apoptomirs between mutant and normal retinas at different ages.** FC differences of apoptomirs as measured by qRT-PCR are shown at 3, 5, 7, 16 wks in xlpra2 and rcd1, as well as 6.4, 8.3-9.9, 11.9-14.1 wks in erd compared to normals. Selected apoptomirs belong to different functional groups: **A)** anti-apoptotic that were DE at later ages: miR-9, -19a, -20; **B)** anti-apoptotic that were DE during the course of disease: miR-21, -155, -221; **C)** pro-apoptotic: miR-122, -129; **D)** dual properties, anti- and pro-apoptotic: miR-29b, -146a. An asterisk indicates statistical significance (p < 0.05; FC > +/-2); bars show SD of biological triplicates. Results for miR-183 are not illustrated because they were not significant in any of the diseases and ages tested.

With the exception of miR-183, all the tested anti-apoptotic apoptomirs were up-regulated in xlpra2 at 16 wks, and miR-155 also at 7 wks (Figure 4A and B). In contrast, the pro-apoptotic apoptomirs (-122, -129) were down-regulated in xlpra2 at 16 wks, the time period after the peak of cell death. The expression pattern of miR-122 was unique; it was down-regulated at 3 wks and up-regulated at 7 wks in xlpra2 (Figure 4C). The apoptomirs with dual anti- and pro-apoptotic properties (miR-29b and -146a) were up-regulated in xlpra2 at 16 wks (Figure 4D). Comparative results between xlpra2 and normal retinas showed high concordance between the hybridization-based microarrays and the amplification-based technology (qRT-PCR) and comparable FC differences in expression were also observed. The only exception was miR-122, which, as described above, reached statistical significance only in the qRT-PCR analysis. Overall, our results suggest that up-regulation of anti-apoptotic and down-regulation of pro-apoptotic miRNAs accompany disease progression in the xlpra2-mutant retinas.

Similar patterns of apoptomir expression were observed in rcd1 and erd retinas, especially at 16 wks, and the magnitude and time course directly reflected the severity and rate of progression of the diseases (Figure 4). Few apoptomirs were DE at early ages in rcd1; i.e. the anti-apoptotic miR-20 and the pro-apoptotic miR-122 were down-regulated at 3 wks, while the anti-apoptotic miR-155 and -221 were up-regulated at 5 wks during the *execution* phase of the disease. At 7 wks, up-regulation was found for miR-155 and -21 in both rcd1 and erd, miR-9, -146a, -221 only in rcd1, and miR-19a, -29b only in erd. In agreement with the results observed in xlpra2, notable results for the rcd1 and erd at 16 wks included down-regulation of the pro-apoptotic miR-122 and -129, and up-regulation of almost all anti-apoptotic miRNAs (Figure 4). The only exception was that the expression of miR-221 was not altered in erd but was highly up-regulated in the other 2 diseases. These results indicate that the expression of the selected apoptomirs is similar in the 3 early-onset canine models studied and suggest that up-regulation of anti-apoptotic and down-regulation of pro-apoptotic miRNAs may be engaged to counteract the PR degeneration process.

#### Expression changes of apoptomirs in the slowly progressive prcd disease

**Figure 5:**
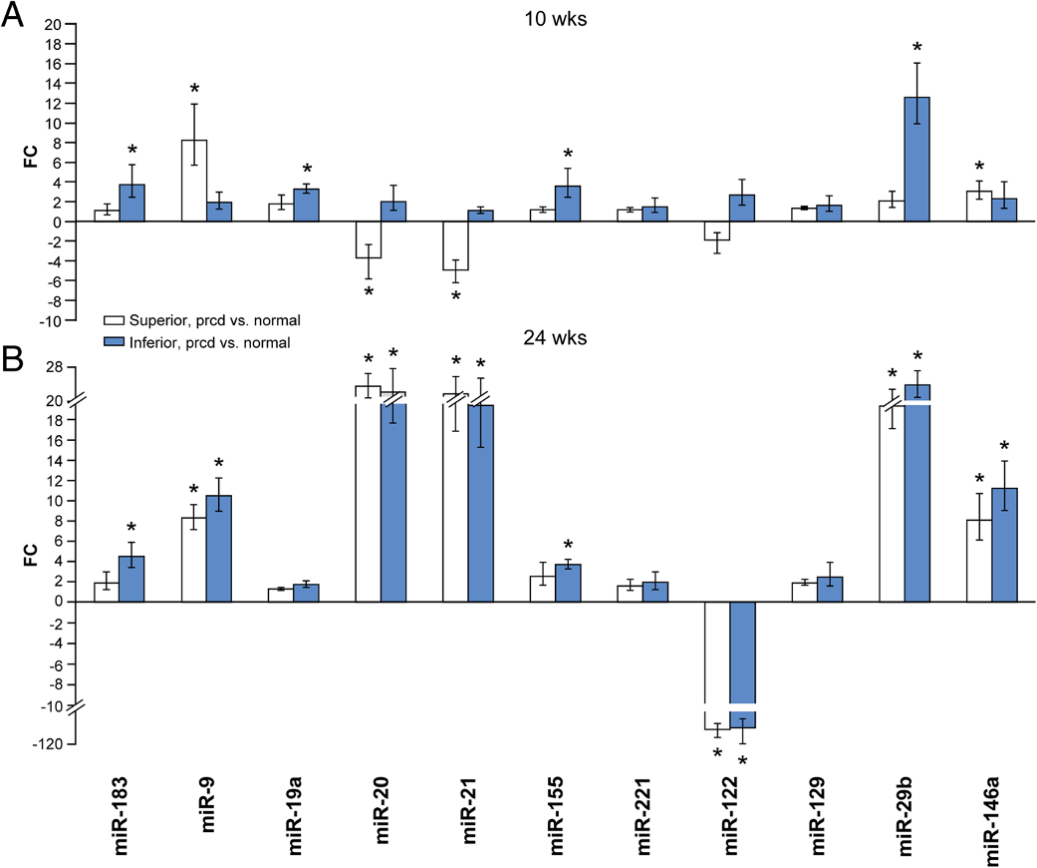
**Expression changes of apoptomirs in prcd vs. normal retinas.** Values that significantly differ as measured by qRT-PCR are indicated with an asterisk (*: p < 0.05; FC > +/-2). **A)** FC differences between either superior or inferior retinas in 10 wks old prcd vs. age and retinal location matched normals. **B)** FC differences between either superior or inferior retinas in 24 wks old prcd vs. 16 wks old entire normal retinas.

To determine if the observed changes in apoptomir expression were specific for early-onset diseases, we analyzed their expression in prcd, a slowly progressive autosomal recessive retinal disorder. The qRT-PCR results showed that 4 miRNAs (up-regulated: miR-9 and -146a; down-regulated: miR-20 and -21) were DE between 10 wks old prcd superior vs. normal retinas and that 4 miRNAs (-19a, -29b, -155, -183) were up-regulated in 10 wks old prcd inferior vs. normals (Figure 5A). These findings indicate that DE miRNAs in prcd at the early age were region-specific as none of them was DE in both superior and inferior retinas.

An increased number of DE apoptomirs was found in 24 wks old prcd vs. 16 wks normals; eight (up-regulated: miR-9, -20, -21, -29b, -146a, -155, -183; down-regulated: -122) in the inferior and six (up-regulated: miR-9, -20, -21, -29b, -146a; down-regulated: -122) in the superior retina (Figure 5B). In contrast to the 10 wk time point, the data at 24 wks demonstrated a similar pattern of expression in superior and inferior retinas indicating a region-independent involvement of apoptomirs at this later age.

Several similarities in miRNA expression patterns were found between prcd and the 3 early-onset diseases. At 10 wks, before PR cell death, DE apoptomirs in the superior prcd retina were the same as in rcd1 at 7 wks and, with the exception of miR-183, the DE apoptomirs in the inferior retina were the same as in erd at 8.3-9.9 wks. At 24 wks, when PRs start to die, DE apoptomirs in the superior and inferior retinas were also DE in xlpra2, rcd1, and erd at 16 wks, with the exception of miR-21 that was not DE in rcd1 (Figure 4). Although prcd is slowly progressive and the phenotype is different from the 3 early-onset diseases, the expression profiles of the selected apoptomirs at later ages were similar. Thus, a common reactive response that causes up-regulation of anti-apoptotic and down-regulation of pro-apoptotic apoptomirs appears to be engaged by the PR degeneration process in all 4 models.

#### Expression changes of apoptomirs in RPE/choroid samples

**Figure 6:**
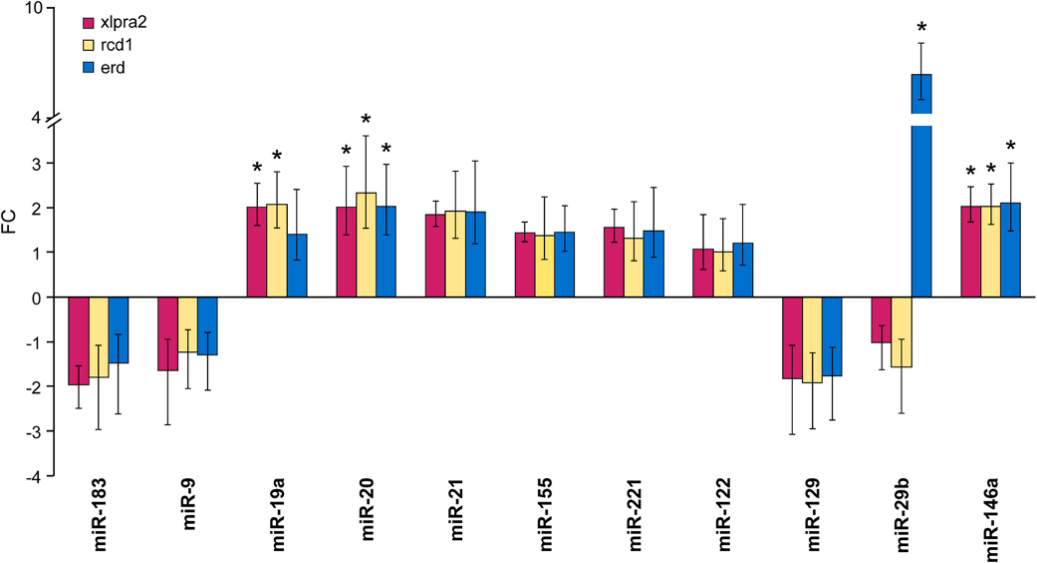
**Expression changes of apoptomirs between mutant and normal RPE/choroids.** FC differences of apoptomirs as measured by qRT-PCR are shown between xlpra2, rcd1, and erd-mutants compared to normals at 7 wks of age. Bars show SD of biological triplicates and values that significantly differ are indicated with an asterisk (*: p < 0.05; FC > +/-2).

The RPE cell layer nourishes the adjacent retinal visual cells and, among other functions, serves to transport small molecules to maintain retinal environment (reviewed by [34]). Although the RPE cells do not degenerate in these canine models until many years after the disease onset, we aimed to determine if the observed expression changes in apoptomirs were specific to the retina or also were present in the RPE. To this end, we compared normal, xlpra2, rcd1, and erd RPE/choroid samples at 7 wks. The results revealed that miR-20 and -146a were up-regulated in all 3 diseases, miR-19a in xlpra2 and rcd1, while miR-29b exclusively in erd (Figure 6). Of these, only miR-146a in rcd1 and -29b in erd were also DE in retina at the same age (Figure 4). These results indicate specific differences in apoptomir expression at 7 wks in the 2 different cell types, and do not suggest a key role of RPE cells at this age in the transport of apoptomirs to the diseased retinas.

### qRT-PCR analysis of genes involved in miRNA biogenesis

To determine if the miRNA expression changes correlated with dysregulation of the entire miRNA machinery, we also examined the age-related expression of *DICER1*, *XPO5*, and *DROSHA*, three genes that are involved in miRNA biogenesis. *DICER1* is also a component of IPA network #3, which was affected by the DE miRNAs at 16 wks (Additional file 3). The qRT-PCR results showed little difference in expression; the miRNA processing enzyme *DROSHA* was down-regulated in erd retinas at 11.9/14.1 wks and at 7 wks in RPE/choroids, while *DICER1* was down-regulated in erd RPE/choroids at 7 wks. As no differences were found between normal and rcd1 or xlpra2-mutants at 3, 5, 7, and 16 wks, or normal and prcd at 10 and 24 wks, our results support a specific dysregulation of miRNA biogenesis in erd-mutants at later ages.

## Discussion

### miRNA expression profiles in normal and xlpra2 retinas

The ability to monitor significant changes in a large number of miRNAs simultaneously is a key factor in understanding their function during aging, and in health and disease. This is particularly relevant, as populations of small RNAs have been shown recently to be extremely complex in mouse retina and RPE/choroid cells [19]. We used the microarray technology to expand our knowledge of miRNA-related mechanisms involved in normal PR development and degeneration in xlpra2 retinas at three key time points previously established for the disease [24].

In normal retina development, miRNA-related changes predominantly occurred between 7–3 wks, with only minimal changes found at later time points when the retina completes development (7 wks) and is structurally and functionally mature (16 wks) [26]. In xlpra2, miRNA developmental expression patterns differed, with fewer DE miRNAs between 7–3 wks and an increased number between 16–7 wks, suggesting that the altered expression at later ages is directly related to disease progression.

These results were confirmed by a direct comparison of the expression profiles of xlpra2 and normal retinas. No DE miRNAs were found at the *induction* phase of the disease (3 wks), when the mutant PRs are developing albeit abnormally, only 2 at the *execution* phase (7 wks), and as many as 173 at the c*hronic cell death* phase (16 wks). The high number of DE miRNAs identified at this later time point suggests that the observed PR degeneration in our canine model elicits major changes in miRNA expression and that these molecules might play a key role late in disease progression. Similar to studies in mice [35, 36], our results also showed unique patterns of miRNA expression changes that were age- and disease stage-dependent. This indicates that DE miRNAs likely have specific functions at different time points in the disease process, and that miRNA-dependent mechanisms triggered during the *chronic cell death* phase of the disease are different from those induced during the *execution* phase.

The observed increase in miR-1 expression was also previously found in the P347S-RHO model [37], rho knockout, D307-rds, and rds null mutants [38] at comparable disease stages. We did not find any changes in expression of either miR-24a, shown to repress apoptosis in the developing *Xenopus* retina [39], or the miRNA-183/96/182 cluster, which is highly expressed in mouse retina and RPE/choroid cells [19] and PR, retinal bipolar, and amacrine cells [35]. This cluster protects the retina from bright light-induced degeneration [40] and syndromic retinal degeneration [41], and is decreased in retinas of transgenic P347S-RHO mice [37]. Although it is difficult to directly compare these results due to differences in experimental conditions, our results indicate that miRNA profiles can be quite similar in different retinal diseases, although model, age, and species specific expression changes also occur.

### Target genes of DE miRNAs

Using a bioinformatics approach, we predicted potential common target genes for the up-regulated (FC > 5) miRNAs in xlpra2 at 16 wks. We identified a total of 35 genes, the function of several of which might be related to PR degeneration. SNTB2 is necessary for eye development in Drosophila [42], SLC1A2 is a glutamate transporter and glutamate reduction was observed in Müller cell in rd1 retina [43], and CDK6 is involved in retina degeneration in mice [44]. While down-regulation of CREB1 has been related to PR cell death in mouse models of retinal degeneration [8], an increase in the levels of native CREB1 has been reported in the rcd1 dog [45]. In addition, phosphorylation of CREB1/ATF1 in PRs of human AMD retinas and in those of canine RP models, including rcd1, erd, and prcd during the *chronic phase* of cell death may contribute to a pro-survival response [45]. These target transcript predictions are useful in highlighting the possible miRNA-dependent regulatory mechanisms that underlie retinal degeneration in the xlpra2-mutant dogs. However, additional experimental studies (for a review see [46]) will be required to validate the predicted miRNA target genes and to determine the effect of these miRNAs on the potential targets in retina.

We previously identified 18 down-regulated transcripts in xlpra2-mutants at 16 wks of age using custom made retina-specific microarrays [47]. None of these genes were among the common 35 predicted targets found in this study at the same age. This could be due to the particular composition of the microarrays (e.g. limited availability and retina-specificity of the genes analyzed), as well as the low number of DE transcripts found.

### Network and functional IPA analyses of DE miRNAs

The IPA software was used to further characterize the changes in miRNA expression at 16 wks. The results indicated an alteration of networks related to the inflammatory response and to cell death and survival. Inflammation accompanies many retina degenerative diseases (reviewed by [32]), including the rd10 mice model of retinitis pigmentosa [31]. In the xlpra2 model, retinal inflammation occurs early during the disease process, and may consequently influence the expression of correlated miRNAs.

Several pathways have been related to PR cell death and survival [5, 9], thus the association of cell death and survival with the observed miRNA signature is particularly provocative. However, since >70% of vertebrate miRNAs are predicted to have at least one target related to cell death/survival, and a single miRNA might regulate a mixture of anti- and pro-apoptotic genes, one must be cautious when categorizing a miRNA by its role in apoptosis (reviewed by [11]).

### Apoptomir expression in normal, xlpra2, rcd1, erd, and prcd

With this caveat in mind, we selected apoptosis and inflammation related apoptomirs for qRT-PCR to confirm the microarray data and expand the results to 3 additional retinal diseases and cell types (i.e. RPE/choroid). The selected apoptomirs are all expressed in retina and were divided into anti-, pro-, or both properties according to the current literature (Additional file 7). Although this approach does not provide a comprehensive miRNA profiling in the additional diseases, the results gives interesting insights and comparison data to better understand the miRNA-related mechanisms in different canine models.

Apoptomirs regulation during normal canine development was in agreement with studies in other species. We found an increased expression of miR-122 at 16 vs. 3 wks, in agreement with studies in normal adult mice compared to postnatal at day 4 [48]. Our data also showed high expression levels of miR-155 early in development, and low expression of miR-19a and -20 after development is completed. miR-155 was highly expressed at early developmental stages in *Xenopus* retina [49] and not detected in 3 month old C57BL/6 J mouse retinas [19], while during murine development miR-19a and -20 are highly expressed at early proliferative stages, but barely detectable in adult retina [50]. These results indicate that the selected apoptomirs exert a common role during normal retina development in several species.

The qRT-PCR results also revealed similar patterns of apoptomir expression at 16 wks in the 3 early-onset models and at 24 wks in prcd, independent of the retinal region. There is a differential rate of degeneration between superior and inferior quadrants in prcd, with the inferior one occurring earlier and being more severe. As the change from disease to degeneration occurs at >25 wks of age [51, 52], our results suggest that disease is comparable between superior and inferior quadrants in the 24 wks old retinas analyzed. The similarities in expression with the other diseases are surprising, as prcd is a slowly progressive disease and no peak of PR death is observed. Although this might be due to low expression levels in normals, these results provide interesting and unexpected commonalities in the expression of some apoptomirs in the canine models that are independent of the phenotype and kinetics of disease. Further studies with an increased number of miRNAs (e.g. microarrays or quantitative next generation sequencing) will be helpful to confirm these initial observations.

The most relevant qRT-PCR finding showed a specific increase in anti-apoptotic and decrease of pro-apoptotic apoptomir expression in all disease models, suggesting that during the *chronic cell death* phase compensatory mechanisms are activated in the mutant retinas in an attempt to prevent PR cell death. While these mechanisms appear insufficient to stop the degenerative process, they may influence the rate of progression. Expression of additional DE miRNAs identified by microarray analysis reinforced this hypothesis: the anti-apoptotic miR-146b, -148a, and -7 were up-regulated in xlpra2, while the pro-apoptotic miR-34b was down-regulated. However, a few exceptions were found; the normally pro-apoptotic let-7 family, miR-15a, and -16 were up-regulated, whereas the anti-apoptotic miR-210 was slightly down-regulated. This might indicate that these miRNAs exert a different function or are involved in the repression of different genes in retina cells. Further analyses are needed to confirm this prediction.

We found up-regulation of miR-29b in old compared to young normal retinas and in mutants vs. normals during the *chronic cell death* phase. This was in agreement with expression pattern in normal mouse retina and in *Nrl*^-/-^ retinas compared to wild type [53]. We also found an increased expression of miR-146a, -155, -9, -21 in mutants. Up-regulation of miR-146a, -155, and -9 was found in age-related macular degeneration [54], while miR-146a, -155, and -21 were up-regulated in P347S-RHO mutants [37]. Yet, the expression pattern of miR-29b and -146a, the apoptomirs with known dual anti- and pro-apoptotic properties, was similar to that of anti-apoptotic miRNAs suggesting that in retinal diseases these two miRNAs might be involved in apoptosis repression.

### Expression of genes involved in miRNA biogenesis

Our results indicate that retinas and RPE/choroids of xlpra2, rcd1, and prcd-mutants have normal expression patterns of effectors required for miRNA biogenesis, suggesting that the miRNA production machinery is not directly responsible for the alteration of the miRNA profiles. In contrast, *DROSHA* was down-regulated in both the retina and RPE/choroids and *DICER1* in RPE/choroids of erd mutants, indicating a dysfunctional miRNA metabolism. Notably, conditional DICER deletion studies in the mouse visual system lead to multiple retinal phenotypes, including increased apoptosis and impaired retinal development, differentiation, and maintenance (reviewed by [16]). The relevance of these observations to the erd model, which showed particular disease-specific features such as concomitant PR cell death and proliferation with formation of hybrid rod/S-cones [27], needs further examination.

## Conclusions

In the current study we found a number of DE miRNAs at the late stage of the xlpra2 disease. We then confirmed differential expression of selected apoptosis-related miRNAs by qRT-PCR and found a similar pattern of expression in rcd1 and to a lesser extent in erd and prcd. These results showed a general up-regulation of anti-apoptotic and down-regulation of pro-apoptotic miRNAs, suggesting that these miRNAs might be engaged to counteract the degenerative processes. Although different mutations trigger the retinal diseases studied, we observed commonalities in the miRNA expression pattern that appear to be associated with the PR cell death kinetics. These findings are highly significant as they suggest that the use of miRNAs as targets for future therapeutic design might be effective in treating the chronic slow cell death phase of retinal degenerative diseases regardless of the initiating mutation.

## Methods

### Tissue samples

Retinal tissues were collected from age-matched normal and mutant dogs under deep pentobarbital anesthesia, and the dogs were then euthanatized. The dogs are maintained at the Retinal Disease Studies Facility in Kennett Square, Pennsylvania, and have a common genetic background but differ primarily at the investigated retinal disease locus [4]. To avoid fluctuations in gene expression with time of the day [55], eyes were collected at a single time period (noon) as previously reported [47]. The research was conducted in full compliance and strict accordance with the Association for Research in Vision and Ophthalmology (ARVO) Resolution on the Use of Animals in Ophthalmic and Vision Research, and all protocols were approved by the University of Pennsylvania Institutional Animal Care and Use Committee (IACUC). All efforts were made to minimize suffering.

### Retinal diseases

Four different canine models were studied: **a**) X-linked progressive retinal atrophy 2 (xlpra2) is the dog homolog of X-linked retinitis pigmentosa (XLRP). The disease is early-onset, affects rods and cones, and is caused by a 2-bp microdeletion in *RPGR* exon ORF15 that creates a frameshift and premature stop in the translated protein [56]. Although the function of RPGR is not yet entirely understood, it has been shown that the protein localizes to the connecting cilium and participates in intraflagellar protein transport, being essential for PR viability and ciliogenesis (reviewed by [57]; **b)** rod cone dysplasia 1 (rcdl) is an early-onset, autosomal recessive rod disease caused by a nonsense mutation in the rod cyclic GMP phosphodiesterase 6 β subunit (*PDE6B*) that results in a stop codon and truncation of the protein by 49 aa [58, 59]. Cone PRs are not affected by the mutation, but also degenerate secondarily; **c)** early retinal degeneration (erd) results from a mutation in *STK38L* that appears to play a role in early PR development [27, 60]. Abnormal development and degeneration of rods and cones characterize the disease and, as an unique feature, concurrent PR apoptosis or mitosis, and formation of hybrid rod/S-cone cells occur [27]. STK38L function in PRs is currently unknown, but recent *in vitro* studies indicate that STK38L-mediated Rabin8 phosphorylation is crucial for ciliogenesis [61]; **d)** prcd is a post-developmental, slowly progressive autosomal recessive disorder [29]. The function of the mutant gene *PRCD* is still unknown. Unlike the other three diseases, prcd is characterized by a topographically distinct pattern of disease distribution. Early and mild PR outer segment disease is present uniformly across the retina at 10 wks of age, but degeneration begins in the inferior retina after 25 wks of age and progresses more rapidly [51, 52]. To address these topographic differences, the globes from prcd-affected dogs were hemisected in the horizontal plane and the superior and inferior retina was isolated and analyzed separately.

### RNA extraction

Total RNA from neuroretina and retinal pigment epithelium (RPE)/choroid was extracted following standard TRIzol procedures (Invitrogen-Life Technologies, Carlsbad, CA). RNA concentration was assessed with a ND-1000 Spectrophotometer® (NanoDrop Technologies, Thermo Fisher Scientific, Wilmington, DE), and RNA quality verified by microcapillary electrophoresis on an Agilent 2100 Bioanalyzer with RNA 6000 Nanochips (Agilent Technologies, Santa Clara, CA). Only high quality RNA with RIN >7 and A260/280 ratio >1.9 was used in both microarray and qRT-PCR analyses.

### Experimental time points and microarray analyses

We initially compared the miRNA expression profiles of normal and xlpra2 dog retinas at 3, 7, and 16 wks, which are crucial ages in the progression of this disease [24]. A minimum of 3 biological replicates/age/group were analyzed [except for normals (3 and 16 wks) and mutant (7 wks) where 4 biological samples/age were used] with miRNA-specific Affymetrix microarrays (GeneChip miRNA Array) containing a total of 46,228 probes, 7,815 probe sets among which 177 are canine specific miRNAs.

### Microarray target preparation and hybridization

Microarray services were provided by the Penn Microarray Facility. All protocols were conducted as described in the standard Affymetrix Expression Analysis Technical Manual (Affymetrix Inc., Santa Clara, CA). Briefly, biotinylated cRNA was prepared from 100 ng total RNA; following fragmentation, cRNA was hybridized for 16 h at 45°C on the Affymetrix miRNA-specific arrays. Microarrays were then washed at low (6X SSPE) and high (100 mM MES, 0.1 M NaCl) stringency and stained with streptavidin-phycoerythrin in an Affymetrix Fluidics Station 400. Fluorescence was amplified by adding biotinylated anti-streptavidin and an additional aliquot of streptavidin-phycoerythrin stain. A confocal scanner (Hewlett-Packard Gene Array Scanner G2500A) was used to collect fluorescence signal after excitation at 570 nm.

### Bioinformatic analyses

Affymetrix command console and expression console were used to quantitate expression levels for targeted miRNAs; default values provided by Affymetrix were applied to all analysis parameters. Border pixels were removed, and the average intensity of pixels within the 75th percentile was computed for each probe. The average of the lowest 2% of probe intensities occurring in each of 16 microarray sectors was set as background and subtracted from all features in that sector. Probe sets for positive and negative controls were examined in expression console, and facility quality control parameters were confirmed to fall within normal ranges. Probes for each targeted miRNA were averaged, log transformed, and inter-array normalization performed using the Robust Multichip Analysis (RMA) algorithm. Unsupervised hierarchical clustering by sample was performed to confirm that replicates within each condition grouped with most similarity, and to identify any outlier samples. A two-way ANOVA with Benjamini-Hochberg (BH)*-*adjusted p < 0.05 and fold change (FC) > +/-2 was applied to generate lists of statistically significant DE miRNAs in pairwise comparisons of replicate averages between conditions. On the Affymetrix microarrays there are several probes for the same miRNA that have the same exact sequence, yet a different nomenclature (different species name) (e.g. cfa-miR-155, hsa-miR-155, bta-miR-155, mml-miR-155, …). Thus, when a miRNA (e.g. -155) represented by several identical probes was DE, we report in the Results section by order of priority the canine (cfa) probe, then the human (hsa). If none of these were represented, we list the one with the species name that has the highest FC difference.

The Ingenuity Pathways Analysis (IPA) database and web-based analysis software (Ingenuity Systems, Inc., Redwood City, CA) [62] was used to identify networks, biological functions, and functional processes that were most significantly associated with the set of DE miRNAs at 16 wks of age.

Furthermore, a target prediction software available online [63] was utilized to predict possible common targets of the up-regulated (FC > 5) miRNAs at 16 wks that had homologues in humans. This comprehensive resource of miRNA target predictions is a development of the miRanda algorithm and uses a compendium of mammalian miRNAs and the mirSVR regression method for predicting likelihood of target mRNAs [64].

### Quantitative real-time PCR (qRT-PCR)

qRT-PCR was used to validate the microarray results of 9 DE apoptomirs, as well as miR-122 and -183, in xlpra2 and normal retinas at 3, 7, and 16 wks. These analyses also included the 5 wk time period in both groups (3 dogs/age/group). The studies were extended to three additional diseases: **a)** rcd1 at the same 4 time points; **b)** erd-mutants at 6.4 wks (n = 2) and 8.3-9.9 wks (n = 3) compared to the 7 wks normal and 11.9-14.1 wks (n = 2) compared to the 16 wk old normal; **c)** prcd-mutant inferior and superior retinas at 10 wks compared to inferior and superior retinas of normal dogs at the same age, and, as only minor miRNA expression changes were observed at 24 wks, both 24 wks old inferior and superior prcd retinas were also compared to 16 wks old normal entire retinas (3 dogs/age/group).

Lastly, RPE/choroids of normal, xlpra2, rcd1, and erd dogs at 7 wks were also analyzed to determine if the observed changes were retina-specific or if they also occurred in neighboring cells.

Eleven miRNAs (Additional file 7) were tested by qRT-PCR with either human or mouse TaqMan assays to amplify canine sequences (Applied Biosystems, Foster City, CA). U43 was used as housekeeping miRNA because its expression was uniform in all tested dogs in the microarray and qRT-PCR analyses. To analyze the expression of 4 genes involved in miRNA biogenesis (Additional file 7), RNA samples were treated with RNase-free DNase (Ambion, Austin, TX), and reverse-transcribed using the High Capacity cDNA Reverse Transcription Kit (Applied Biosystems). The real-time reactions included 30 ng of cDNA and canine specific TaqMan probes. Glyceraldehyde 3-phosphate dehydrogenase (*GAPDH*) was used as housekeeping control because in the tested diseases it performed most accurately, and with the least variation between samples [27, 47].

All the qRT-PCR reactions were performed in 96 well plates using an ABI 7500 real-time PCR machine with the 7500 detection software (v2.0.1, Applied Biosystems). Comparisons were performed with the ΔΔCT method [65] and statistical significance was verified with an unpaired t-test (p < 0.05) and FC > +/-2.

## Availability of supporting data

The complete microarray data set supporting the results of this article were deposited in NCBI’s Gene Expression Omnibus [66] following the guidelines of the rationale of minimum information about a microarray experiment (MIAME), and are accessible through GEO Series accession number GSE35205.

## Electronic supplementary material

Additional file 1: **DE miRNAs in normal retinas at different ages.** DE miRNAs (BH-adjusted p < 0.05 and FC > +/-2) identified by microarray analysis in normal retinas at 7 vs. 3, 16 vs. 7, and 16 vs. 3 wks of age. miRNAs are listed from the highest to the lowest fold change difference. In green, apoptomirs tested by qRT-PCR with the corresponding p-value and FC difference. (XLS 64 KB)

Additional file 2: **DE miRNAs in xlpra2-mutant retinas at different ages.** DE miRNAs (BH-adjusted p < 0.05 and FC > +/-2) identified by microarray analysis in xlpra2-mutant retinas at 7 vs. 3, 16 vs. 7, and 16 vs. 3 wks of age. miRNAs are listed from the highest to the lowest fold change difference. In green, apoptomirs tested by qRT-PCR with the corresponding p-value and FC difference. (XLS 61 KB)

Additional file 3: **DE miRNAs between xlpra2 and normal retinas.** DE miRNAs (BH-adjusted p < 0.05 and FC > +/-2) identified by microarray analysis at 7 and 16 wks of age in xlpra2-mutants compared to normals. miRNAs are listed from the highest to the lowest fold change difference. In green, apoptomirs tested by qRT-PCR with the corresponding p-value and FC difference. No expression differences were found at 3 wks. (XLS 84 KB)

Additional file 4: **IPA network “Cancer, Reproductive System Disease, Endocrine System Disorders” significantly affected by DE miRNAs.** Most significantly affected IPA network “Cancer, Reproductive System Disease, Endocrine System Disorders” identified with 23 up-regulated (marked in red) and 3 down-regulated (marked in green) miRNAs that were DE by microarray analysis at 16 wks between xlpra2 and normal retinas. The complete list of miRNAs and genes belonging to this network and the additional 6 significantly affected networks are detailed in Additional file 5. The figure was adapted from Ingenuity Systems [62]. (TIFF 1017 KB)

Additional file 5: **IPA networks associated with the DE miRNAs at 16 wks.** The 7 IPA affected networks that were associated with the DE miRNAs identified at 16 wks between xlpra2 and normal retinas are listed with the number (in bold in red up- and green down-regulated), all node molecules that belong to the network, and the corresponding main functions. A graphical representation of network 1 is shown in Additional file 4. (XLS 33 KB)

Additional file 6: **IPA biological functions associated with the DE miRNAs at 16 wks.** The IPA five most affected biological functions (p < 0.05) that belong to the categories “Diseases and Disorders” and “Molecular and Cellular Functions” are reported. The biological functions are shown with the affected sub-groups (from the lowest to the highest p-values), p-values, and the corresponding DE miRNAs. (XLS 117 KB)

Additional file 7: **qRT-PCR assays used to examine the expression of miRNAs or genes involved in miRNA biogenesis.** The miRNA and gene specific TaqMan expression assays (Applied Biosystems catalog #) used for qRT-PCR are reported with the currently known apoptosis-related function and selected references [11, 16, 40, 67–87]. (DOCX 16 KB)

## Abbreviations

AMD: Age-related macular degeneration
Apoptomirs: Apoptosis-related miRNAs
ARVO: Association for Research in Vision and Ophthalmology
ATF1: Activating transcription factor 1
BH: Benjamini Hochberg
BIRC5: Baculoviral IAP repeat containing 5
CDK6: Cyclin-dependent kinase 6
CREB1: cAMP responsive element binding protein 1
DE: Differentially expressed
erd: Early retina degeneration
DNAH14: Dynein, axonemal, heavy chain 14
ERG: Electroretinography
E2F1: E2F transcription factor 1
FC: Fold change
GAPDH: Glyceraldehyde 3-phosphate dehydrogenase
IACUC: Institutional Animal Care and Use Committee
IL21: Interleukin 21
IPA: Ingenuity Pathway Analysis
KSR2: Kinase suppressor of ras 2
MIAME: Minimum information about a microarray experiment
miRNA and miR: microRNA
NFkB: Nuclear factor of kappa light polypeptide gene enhancer in B-cells
PAG1: Phosphoprotein associated with glycosphingolipid microdomains 1
PARP: Poly (ADP-ribose) polymerase family
PAX6: Paired box 6
PDE6B: Rod cyclic GMP phosphodiesterase 6 β subunit
PR: Photoreceptor
prcd: Progressive rod-cone degeneration
qRT-PCR: Quantitative real-time PCR
rcd1: Rod cone dysplasia 1
RIN: RNA integrity number
RMA: Robust Multichip Analysis
RPE: Retinal pigment epithelium
RPGR: Retinitis pigmentosa GTPase regulator
SCAI: Suppressor of cancer cell invasion
SD: Standard deviation
SIRT1: Sirtuin 1
SLC1A2: Solute carrier family 1 (glial high affinity glutamate transporter) member 2
SNTB2: Syntrophin beta 2
STK38L: Serine/threonine kinase 38 like
Vegf: Vascular endothelial growth factor
VIM: Vimentin
wks: Weeks
xlpra2: X-linked progressive retinal atrophy 2
XLRP: X-linked retinitis pigmentosa
XPO5: Exportin 5.
