# Supplementary figures and images for: Altered miRNA expression in canine retinas during normal development and in models of retinal degeneration
Source: BMC Genomics. 2014 Mar 1;15(1):172. doi: 10.1186/1471-2164-15-172 (PMC4029133; doi:10.1186/1471-2164-15-172)

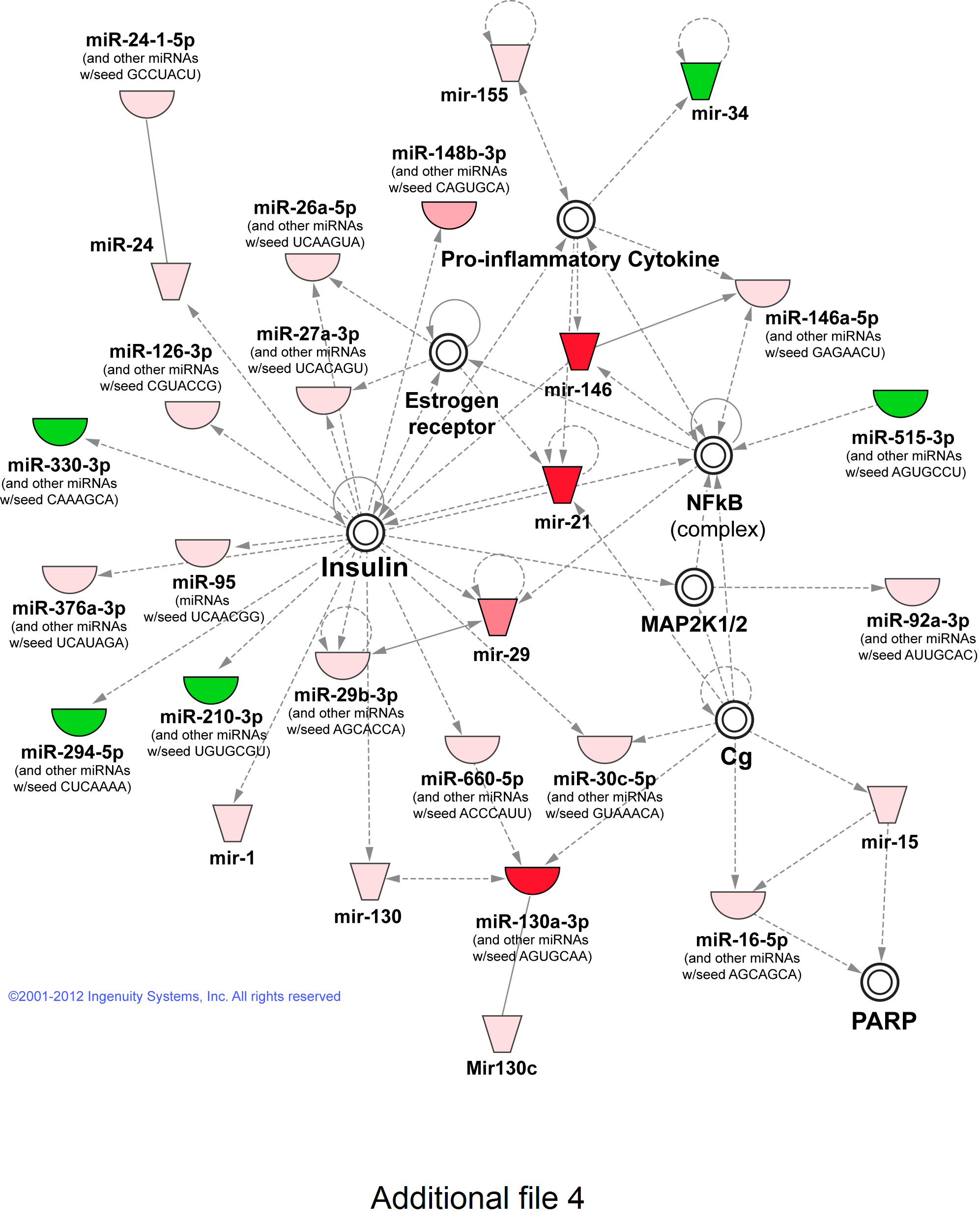

Supplement: Supplementary file 4 — Additional file 4: IPA network “Cancer, Reproductive System Disease, Endocrine System Disorders” significantly affected by DE miRNAs. Most significantly affected IPA network “Cancer, Reproductive System Disease, Endocrine System Disorders” identified with 23 up-regulated (marked in red) and 3 down-regulated (marked in green) miRNAs that were DE by microarray analysis at 16 wks between xlpra2 and normal retinas. The complete list of miRNAs and genes belonging to this network and the additional 6 significantly affected networks are detailed in Additional file 5. The figure was adapted from Ingenuity Systems [62]. (TIFF 1017 KB) [file 12864_2013_7018_MOESM4_ESM.tiff]
