# Supplementary material for: Altered miRNA expression in canine retinas during normal development and in models of retinal degeneration
Source: BMC Genomics. 2014 Mar 1;15(1):172. doi: 10.1186/1471-2164-15-172 (PMC4029133; doi:10.1186/1471-2164-15-172)
Supplement: Supplementary file 7 — Additional file 7: qRT-PCR assays used to examine the expression of miRNAs or genes involved in miRNA biogenesis. The miRNA and gene specific TaqMan expression assays (Applied Biosystems catalog #) used for qRT-PCR are reported with the currently known apoptosis-related function and selected references [11, 16, 40, 67–87]. (DOCX 16 KB) [file 12864_2013_7018_MOESM7_ESM.docx]

**Additional file 7. qRT-PCR assays used to examine the expression of miRNAs or genes involved in miRNA biogenesis**. The miRNA and gene specific TaqMan expression assays (Applied Biosystems catalog #) used for qRT-PCR are reported with the currently known apoptosis-related function and selected references.

| **miRNA** | **TaqMan assay** | **Relevant apoptosis-related function (selected references)** |
| --- | --- | --- |
| U43 small nuclear RNA | [RNU43](https://products.appliedbiosystems.com:443/ab/en/US/adirect/ab?cmd=ABAssayDetailDisplay&assayID=001095&Fs=y&SearchRequest.Common.PageNumber=1&assayType=taqman&chkBatchQueryText=false&srchType=keyword&searchValue=u43&searchBy=all&msgType=ABmiRNAKeywordResults) (#001095) | housekeeping |
| miR-183 | hsa-miR-183 (#002269) | anti-apoptotic [40,67] |
| miR-9 | hsa-miR-9 (#000583) | anti-apoptotic [68,69] |
| miR-19a | hsa-miR-19a (#000395) | anti-apoptotic [70,71] |
| miR-20 | hsa-miR-20a (#000580) | anti-apoptotic [11,72] |
| miR-21 | hsa-miR-21 (#000397) | anti-apoptotic [11,73] |
| miR-155 | hsa-miR-155 (#002623) | anti-apoptotic [74,75] |
| miR-221 | mmu-miR-221 (#001134) | anti-apoptotic [73,76] |
| miR-122 | hsa-miR-122 (#002245) | pro-apoptotic [77,78] |
| miR-129 | hsa-miR-129 (#000590) | pro-apoptotic [79,80] |
| miR-29b | hsa-miR-29b (#000413) | anti-apoptotic [81,82] and pro-apoptotic [11,83] |
| miR-146a | hsa-miR-146a (#000468) | anti-apoptotic [84,85] and pro-apoptotic [86,87] |
| **Genes involved in miRNA biogenesis** | | Reviewed by [16] |
| *XPO5* (exportin 5) | Cf02638762_m1 | mediates the nuclear export of pre-miRNA hairpins and short hairpin RNAs |
| *Drosha* (ribonuclease type III, nuclear; *RNASEN*) | Cf02687024_m1 | forms the microprocessor complex (with DGCR8) and cleaves pri-miRNA to produce the pre-miRNA, a stem-loop structure of ca. 70 bp |
| *Dicer1* (ribonuclease type III) | Cf02636746_m1 | cleaves the pre-miRNA hairpin yielding an imperfect miRNA:miRNA* duplex of ca. 22 nucleotides |
| *GAPDH* | Hs02786624_g1 | housekeeping |
